# Novel compound heterozygous variants in *XYLT1* gene caused Desbuquois dysplasia type 2 in an aborted fetus: a case report

**DOI:** 10.1186/s12887-022-03132-5

**Published:** 2022-01-26

**Authors:** Fatemeh Rajabi, Ali Hosseini Bereshneh, Mahboubeh Ramezanzadeh, Masoud Garshasbi

**Affiliations:** 1 Department of Medical Genetics, Faculty of Medical Sciences, Tarbiat Modares University, Tehran, Iran; 2 Prenatal Diagnosis and Genetic Research Center, Dastgheib Hospital, Shiraz University of Medical Sciences, Shiraz, Iran; 3 Department of Genetics and Molecular Medicine, Faculty of Medicine, Bushehr University of Medical Sciences, Bushehr, Iran

**Keywords:** Desbuquois dysplasia 2, *XYLT1* mutations, Skeletal dysplasia, Compound heterozygous

## Abstract

**Background:**

Desbuquois dysplasia type 2 (DBQD2) is an infrequent dysplasia with a wide range of symptoms, including facial deformities, growth retardation and short long bones. It is an autosomal recessive disorder caused by mutations in the *XYLT1* gene that encodes xylosyltransferase-1.

**Case presentation:**

We studied an aborted fetus from Iranian non-consanguineous parents who was therapeutically aborted at 19 weeks of gestation. Ultrasound examinations at 18 weeks of gestation revealed growth retardation in her long bones and some facial problems. Whole-exome sequencing was performed on the aborted fetus which revealed compound heterozygous *XYLT1* mutations: c.742G>A; p.(Glu248Lys) and c.1537 C>A; p.(Leu513Met). Sanger sequencing and segregation analysis confirmed the compound heterozygosity of these variants in *XYLT1.*

**Conclusion:**

The c.1537 C>A; p.(Leu513Met) variant has not been reported in any databases so far and therefore is novel. This is the third compound heterozygote report in *XYLT1* and further supports the high heterogeneity of this disease.

**Supplementary Information:**

The online version contains supplementary material available at 10.1186/s12887-022-03132-5.

## Background

Desbuquois dysplasia (DBQD; MIM 615,777) is an autosomal recessive skeletal disorder categorized in a group of dysplasia with multiple joint dislocations. DBQD is a heterogeneous condition that overlaps with other skeletal dysplasia [1, 2]. The clinical manifestations contain severe prenatal and postnatal growth retardation, frailty joint, round face, midface hypoplasia, prominent eyes, short extremities and progressive scoliosis [3]. According to the presence or absence of hand abnormality, this condition divides into two subfamilies including DBQD1 and DBQD2.

In DBQD2, no hand abnormality is observed and it is caused by mutations in the *xylosyltransferase 1* gene (*XYLT1;* MIM 608,124). The DBQD1 is caused by *CANT1* (MIM 613,165) mutations. Some forms of DBQD2 are also caused by mutations in *CANT1* [2, 4, 5]. *XYLT1* gene (NM_022166.4) is located on chromosome 16p12.3 and consists of 12 exons. This gene encodes xylosyltransferase 1 (XT1) (EC 2.4.2.26, NP_071449.1), which is involved in the proteoglycan (PG) synthesis. PG’s structure is made of a core protein with one or more glycosaminoglycan (GAG) chains and XT1 or XT2 transfers xylose from the uridine diphosphate (UDP)-xylose to a specific serine residue of the core protein. [6, 7]. PGs are one of the main parts of the extracellular matrix (ECM). ECM is part of the cell structure and has various biological functions like cellular differentiation and cell growth. Thus, loss of XT protein causes some skeletal dysplasias like osteoarthritis [8].

In this study, we applied whole-exome sequencing (WES) on an aborted fetus, who showed skeletal dysplasia in ultrasound at 18 weeks of gestation, which led to the identification of compound heterozygous variants in *XYLT1*.

## Case presentation

An aborted female fetus around 19 weeks of gestation was referred to the DeNA laboratory, Tehran, Iran. It was the first product of conception for a healthy, Iranian non-consanguineous couple; a 34-year-old mother and a 37-year-old father. Ultrasound at 11 weeks and 6 days of gestation (CRL= 55 mm) reported normal volume of amniotic fluid, nuchal translucency (NT= 1.35 mm) and fetal heart rate (FHR= 171 bmp). The fetus was suspected of dysplasia upon routine prenatal ultrasound evaluation performed at 18 gestation weeks. Ultrasound examination at 18 weeks and 3 days of gestation showed that growth of the femur in the fetus was proportional to < 3rd centile (Z- score: -2.53) and represented 10 days to 2 weeks delay (Table 1). Moreover, the growth in other bones was equivalent to < 3-10th centile, and one-week growth retardation was seen in the tibia, humerus and ulna bones (Table 1). Her head circumference (HC) was 156 mm (63th centile) and her abdominal circumference (AC) was equal to 132.7 mm (56th centile). In addition, the proband had facial characteristics such as frontal bossing, nasal bridge depression, slight midface hypoplasia, small nasal bone (25th centile) and slight brachycephalic. The other biometric parameters including weight (225 gr; 50th centile), amniotic fluid index (7 cm) and nuchal fold sickness (2 mm) were normal. Based on the sonographic findings the fetus was therapeutically aborted.

**Table 1:** Fetal body parameters in 18 weeks + 5 days of gestation by ultrasound

Fibula length	TL	RL	UL	HL	BPD	HC	AC	FL
20.5 mm Z-score: -1.47	21 mm Z-score: -1.47	21 mm Z-score: -0.92	21mm Z-score: -1.94	23 mm Z-score: -2.06	41 mm Z-score: +0.3	150 mm Z-score: +0.4	132.mm Z-score: +0.34	22 mm Z-score: -2.51
*Abbreviations: BPD* biparietal diameter; *FL* femur length; *HC* head circumference; *HL* humerus length; *AC* abdominal circumference; *TL* Tibia length; *UL* Ulna length; *RL* Radius length								

Whole-exome sequencing was performed on the affected fetus using the Nextera Rapid Capture Exome kit which consists of more than 340,000 unique probes to cover 214,405 exons of all human genome chromosomes. The generated library was sequenced on a HiSeq 4000, Illumina (Illumina, Inc., San Diego, CA, USA) with an average coverage depth of 111X. All disease-causing variants reported in HGMD® and ClinVar as well as the variants with minor allele frequency [MAF] of less than 1% in the ExAc database were considered. The evaluation was focused on exons and intron boundaries $\pm$20. Burrows-Wheeler Aligner software (version 0.7.15-r1140), Genome Analysis Toolkit [GATK] were used for aligning the reads to the reference genome GRCh37 [hg19]. Variant calling, annotation and filtering were performed as previously described [9] and revealed two previously unreported compound heterozygous *XYLT1* variants: c.742G>A; p.(Glu248Lys); Chr16 (GRCh37): g.17,353,016 C>T and c.1537 C>A; p.(Leu513Met); Chr16 (GRCh37): g.17235060G>T in the fetus. The c.1537 C>A variant has not been reported in any available databases and was classified as a variant with uncertain significance (class 3) according to the ACMG recommendations. However, the c.742G>A variant (rs765052371*)* had been reported previously with the allele frequency 0.00009 (ALFA Project). In order to confirm these two missense point mutations in the fetus, Sanger sequencing was performed. In this method, the fragments were amplified using PCR and then PCR products were sequenced by ABI 3130XL capillary electrophoresis. The primers were 5′-GGATGTCTGGGTGTGTAGAGG-3′ and 5′-CAGGTTCATTCGGAAGCAGG-3′ (for the c.1537 C>A); and 5′- GAGCAGAATGGGGCTGGG-3′ and 5′- GGTGAGGTGCTGCCTCC-3′ (for the c.742G>A).

**Fig. 1:**
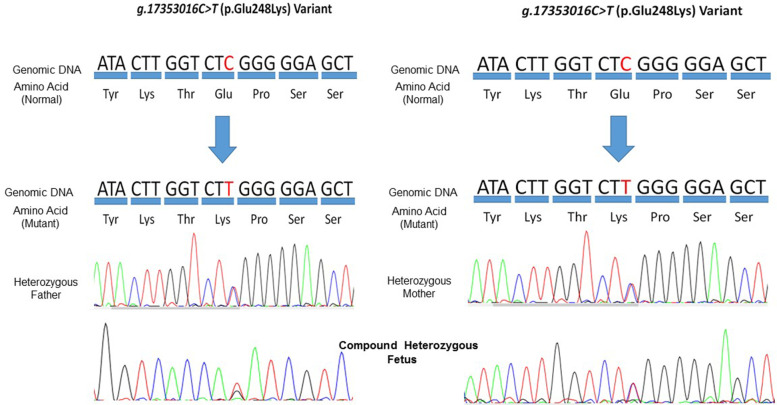
Sanger sequencing and the mutation segregation

**Fig. 2:**
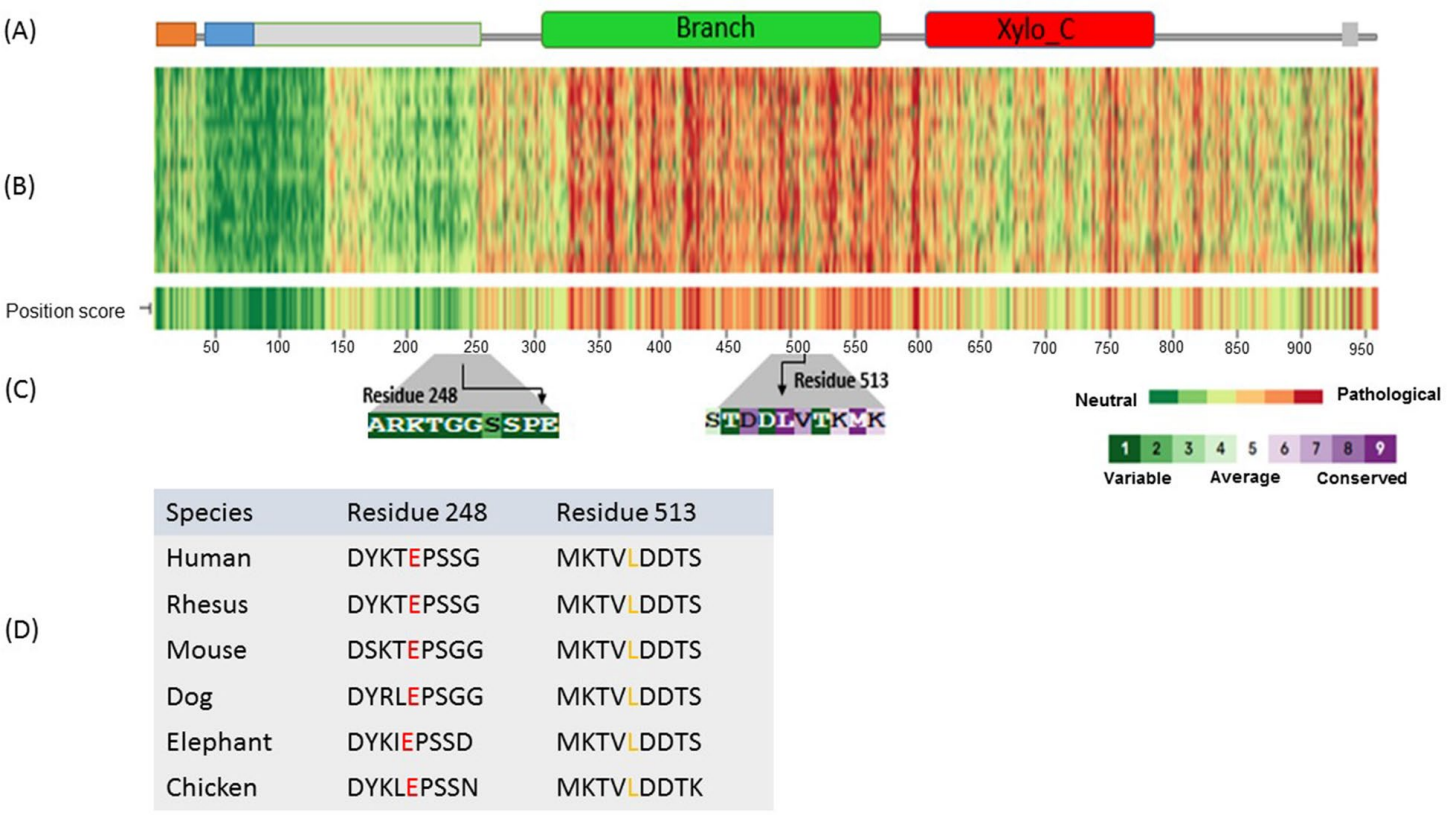
**a** Illustrates the important domains of XT1 protein. **b** The overall view of the protein and conserved domains are illustrated. **c** Conservation scores of the residue 513 of the xylosyltransferase 1 protein analyzed by Consurf server. **d** Conservation of residues 513 and 248 in vertebrates

**Fig. 3:**
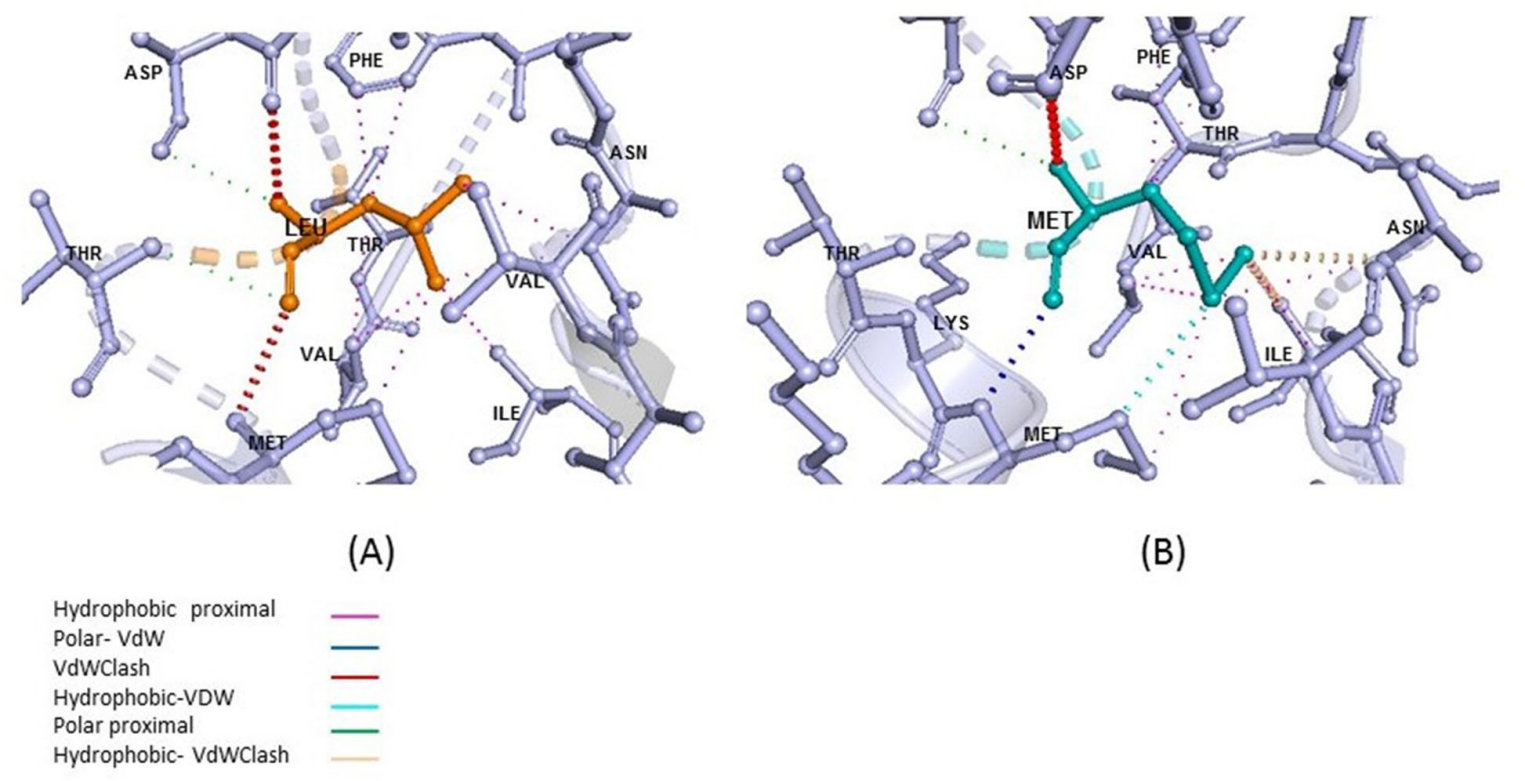
Effect of p.(Leu513Met) mutation on interatomic interactions of XT1 protein. **a** Normal; **b** Mutant. Structural topology has been changed in the mutant form

There was no history of DBQD2 in her family and relatives. Segregation analysis showed that the mother was heterozygous for the c.742 G>A and the father was heterozygous for the c.1537 C>A variant. Hence, this result confirmed compound heterozygosity of the c.742 G>A and c.1537 C>A variants in the *XYLT1* gene (Fig. 1). In order to predict the pathogenicity of the variants, *in silico* prediction of the functional effect of these alternations at the amino-acid levels was done by PolyPhen-2 (http://geneics.bwh.harvard.edu/pph2), SIFT (http://sift.jcvi.org), PMut (http://mmb.irbbarcelona.org/PMut), PROVEAN (http://provean.jcvi.org/index.php) and CADD (https://cadd.gs.washington.edu/) (Supplementary Table 1). XT1 encodes 959 amino acids in the precursor structure that is composed of two domains including the glycosyltransferase family 14 (Core-2/I-Branching enzyme, amino acid 328-581) and xylosyltransferase (Xylo-C, amino acid 613-794) (Fig. 2a). The overall amino acid conservation of XT1 were shown in Fig. 2b. To be more specific, analyzing the conservation of Glu248 and Met513, using ConSurf server (https://consurf.tau.ac.il/), illustrated that Glu248 is not highly conserved unlike Met513 (Fig. 2c). However, amino acid alignment using the UCSC database showed that the 248 and 513 residues are highly conserved in vertebrates (Fig. 2d). E248K alternation is placed in the region of “disorder” and can affect the surrounding amino acids of glutamic acid, including the amino acids serine and threonine. It is predicted that these amino acids are the casein kinase II phosphorylation site and the E248K variant changes this function using different tools such as ScanProsite (https://prosite.expasy.org/scanprosite/). The L513M variant changes a residue inside the glycosyltransferase domain. Most of the reported mutations in this region lead to reduction or loss of enzyme activity according to UniProtKB (https://www.uniprot.org/). Thus, the L513M variant is supposed to reduce the enzyme activity in this case too. The impact of the novel variant on the protein was depicted by using PyMOL [10], Dynamut [11], OCTOPUS [12] and FOLDING RaCe [13]. Study of L513M in the XT1 protein showed this variant does not influence its hydrogenous interactions, even though it affects other interatomic interactions (Fig. 3) and destabilizes (ΔΔG: -0.311 kcal/mol) the protein. Evaluation of L513M showed that the logarithmic change in the folding rate of the protein is equal to -0.41/s. Moreover, the vibrational entropy energy differences between wild-type and mutant (ΔΔS_Vib_ ENCoM= -4.189 kcal.mol-1.K-1) depicted decreased molecule flexibility.

## Discussion and conclusion

The aborted fetuses at 18 weeks of gestation showed short limb bones and facial bone abnormalities on ultrasound. To identify pathogenic variants, we carried out WES, allowing us to identify the genetic cause of DBQD2. Flat face, narrow thorax, low nasal bridge, joint dislocations, Swedish key/monkey wrench appearance, brachymetacarpia and short long bones are the prominent features of the DBQD2 which are mainly observed after birth [14, 15]. Despite this, most cases of DBQD2 show nasal bridge depression and short extremities in ultrasonography during gestational age [7, 16]. The patient in our study indicated relative macrocephaly (HP: 0004482), depressed nasal bridge (HP: 0005280) and short long bone (HP: 0003026) according to Human Phenotype Ontology terms [17] which are comparable with previous reported cases. Molecular studies of the fetus and her parents revealed unreported compound heterozygous variants in *XYLT1* gene. Although various distinct homozygous mutations have been reported in *XYLT1*, only a few compound heterozygous mutations (c.595 C*>*T & c.1651 C*>*T [1] in a Polish patient and c.1588-10_1595del & 3.3 Mb del [6] in a Dutch patient) have been reported. In the Dutch patient who had a compound heterozygote mutations in *XYLT1* gene, the clinical phenotypes that were observed included only short limbs, cleft palate, a short nose with depressed nasal bridge and respiratory problems, without other physical characteristics of DBQD2 [6]. It may indicate that the clinical phenotypes of compound heterozygote mutations are somewhat different from homozygous mutations.

Although various studies have reported different prenatal and postnatal symptoms such as endocrinological problems, in our study, the abortion occurred at 19 weeks which made it impossible to follow the other clinical presentations after birth.

The study by Ranza et al. found that the number of patients with clinical symptoms of DBQD2 had no mutation in the *XYLT1* gene. It reveals that DBQD2 has clinical manifestations that overlap with other different skeletal disorders including Larsen syndrome (MIM 150,250, 245,600, LRS), Spondylo-Epi‐Metaphyseal Dysplasia with Joint Laxity, leptodactylic type (MIM 603,546, SEMDJL2), Desbuquois dysplasia type 1 and Kim variant, Spondylo‐Epiphyseal Dysplasia with dislocations (MIM 143,095) and chondrodysplasia that their pathogenic effects attribute to synthesis or sulfation of proteoglycans (PG) [14]. Another possible explanation could be that DSBQ2 is a heterogeneous disease. Therefore, exome sequencing helps to detect the cause of diseases in cases in which the clinical phenotype indicates one or more syndromes [18].

XT1 and XT2 are type II transmembrane proteins that are included a short amino-terminal region facing the cytosol, a single transmembrane helix and a stem region required for Golgi localization [19]. The variants identified in this study affect amino acid numbers 248 and 513; both of them are part of the luminal topological domain. Mutations in *XYLT1* gene could change the topology and structure of protein and therefore not properly localize into the cell membrane; thus, the transferase activity of the XT1 protein will be disrupted.

This enzyme catalyzes the first step in the biosynthesis of PGs like chondroitin sulfate (CS), dermatan sulfate (DS) and heparan sulfate (HS) proteoglycans [20]. The importance of PGs is in cellular homeostasis and impacting many fundamental biological processes including growth factor function, morphogen gradient formation, co-receptor activity, neuronal regeneration, signaling and development of many tissues in humans [21, 22]. Mizumoto et al. in 2015 showed that the biosynthesis of high-molecular-weight CS-PGs, but not HS-PGs, was less in the cells with *XYLT1* mutations than in healthy controls because these manifestations may be caused by reductions in CS side chains. These findings indicate that XT1 mainly acts on serine residues in the core proteins of CS-PG, but not HS-PG, and the functions of *XYLT1* cannot be compensated by *XYLT2* [23].

In conclusion, we report a new DBQD2 case carrying two novel compound heterozygous mutations, p.(Leu513Met) and p.(Glu248Lys) in *XYLT1*. Desbuquois dysplasia type 2 has vast symptoms and complications. Therefore, due to the variety and severity of complications, identifying and reporting related mutations and using them in prenatal diagnosis is a useful diagnostic tool in the prevention of this disease and benefits children and families affected by this disease.

## Supplementary Information

**Additional file 1.**

## Data Availability

All data generated or analyzed during this study are included in this published article. Data sharing does not apply to this report as no data sets were generated or analyzed.

## Abbreviations

XT1: Xylosyltransferase 1
PG: Proteoglycan
GAG: Glycosaminoglycan
UDP-xylose: Uridine diphosphate-xylose
ECM: Extracellular matrix
WES: Whole-exome sequencing
HC: Head circumference
AC: Abdominal circumference
CD-PGs: Chondroitin sulfate proteoglycans
CS: Chondroitin sulfate
DS: Dermatan sulfate
HS: Heparan sulfate
ER: Endoplasmic reticulum
DBQD: Desbuquois dysplasia
NT: Nuchal translucency
FHR: Fetal heart rate
