# Supplementary material for: Novel compound heterozygous variants in XYLT1 gene caused Desbuquois dysplasia type 2 in an aborted fetus: a case report
Source: BMC Pediatr. 2022 Jan 26;22:63. doi: 10.1186/s12887-022-03132-5 (PMC8790879; doi:10.1186/s12887-022-03132-5)
Supplement: Supplementary file 1 — Additional file 1. [file 12887_2022_3132_MOESM1_ESM.docx]

**Supplementary table 1**. Online database to predict the pathogenicity of E248K and L513M.

| Gene | NM | Exon | Alternation | SIFT | PolyPhen-2 | Mutation Taster | PMut | CADD | PROVEAN |
| --- | --- | --- | --- | --- | --- | --- | --- | --- | --- |
| XYLT1 | NM_022166.4 | 3 | c.742G>A; p.(Glu248Lys) | Tolerated | probably damaging | Damaging | Neutral | Likely Benign | Neutral |
| XYLT1 | NM_022166.4 | 7 | .1537C>A; p.(Leu513Met) | Damaging | probably damaging | damaging | Disease | Uncertain Significance with minor pathogenic evidence | Neutral |
